# The effect of accelerometer location on the classification of single-site forearm mechanomyograms

**DOI:** 10.1186/1475-925X-9-23

**Published:** 2010-06-10

**Authors:** Natasha Alves, Ervin Sejdić, Bhupinder Sahota, Tom Chau

**Affiliations:** 1 Bloorview Research Institute, Bloorview Kids Rehab, Toronto, Ontario, Canada; 2 Institute of Biomaterials and Biomedical Engineering, University of Toronto, Toronto, Ontario, Canada

## Abstract

**Background:**

Recently, pattern recognition methods have been deployed in the classification of multiple activation states from mechanomyogram (MMG) signals for the purpose of controlling switching interfaces. Given the propagative properties of MMG signals, it has been suggested that MMG classification should be robust to changes in sensor placement. Nonetheless, this purported robustness remains speculative to date. This study sought to quantify the change in classification accuracy, if any, when a classifier trained with MMG signals from the muscle belly, is subsequently tested with MMG signals from a nearby location.

**Methods:**

An arrangement of 5 accelerometers was attached to the flexor carpi radialis muscle of 12 able-bodied participants; a reference accelerometer was located over the muscle belly, two peripheral accelerometers were positioned along the muscle's transverse axis and two more were aligned to the muscle's longitudinal axis. Participants performed three classes of muscle activity: wrist flexion, wrist extension and semi-pronation. A collection of time, frequency and time-frequency features were considered and reduced by genetic feature selection. The classifier, trained using features from the reference accelerometer, was tested with signals from the longitudinally and transversally displaced accelerometers.

**Results:**

Classification degradation due to accelerometer displacement was significant for all participants, and showed no consistent trend with the direction of displacement. Further, the displaced accelerometer signals showed task-dependent de-correlations with respect to the reference accelerometer.

**Conclusions:**

These results indicate that MMG signal features vary with spatial location and that accelerometer displacements of only 1-2 cm cause sufficient feature drift to significantly diminish classification accuracy. This finding emphasizes the importance of consistent sensor placement between MMG classifier training and deployment for accurate control of switching interfaces.

## I. Background

The mechanomyogram (MMG) is the superficial vibratory signal measured from contracting muscles. MMG is generated from gross lateral movement of the muscle at the initiation of a contraction, smaller subsequent lateral oscillations at the resonant frequency of the muscle, and dimensional changes of active muscle fibers [1-3]. The interference MMG signal is a compound signal in which active muscle fibre twitches may be summated linearly or non-linearly [4], and is considered an indicator of the activity of individual motor units during voluntary isometric contraction [5]. The MMG signal reflects motor unit recruitment [6], is affected by motor unit fusion [7], and is considered to be a qualitative indicator of the global motor unit firing rate [8]. MMG is measured by microphones [9], piezoelectric contact sensors [10,11], accelerometers [12] or laser distance sensors [13] on the surface of the skin. As the mechanical index of muscle contraction, MMG has been used to study muscle pain [14], muscle fatigue [7,15] and paediatric muscle disease [16]. Recently, MMG has been investigated as a control signal for muscle-driven switching interfaces for applications such as powered upper-limb prostheses [17,18], and alternative access technologies [19,20].

In an early investigation of MMG as a control signal for upper-limb prostheses, Barry et. al [21] used the amplitude of the MMG signal recorded from the belly of a single forearm muscle (wrist flexor or extensor) for tri-state control (flex, extend, rest) of a prosthetic hand. The study suggested that the MMG signal was qualitatively less sensitive to placement on the muscle than electromyography (EMG), and therefore suggested that MMG should be less sensitive to small displacements of the sensor. In recent studies on MMG-driven control, the control state has been identified by classifying MMG signal features recorded from multiple muscle sites [17,20]. As in the case of EMG control [22,23], pattern-recognition-based MMG control assumes that patterns of muscle activity associated with a state of muscle activation are reflected in MMG signal features that are differentiable among different muscle activations states, and repeatable for a given muscle activation state. Each MMG sensor provides localized information about the activity of the underlying muscles, and may be affected by crosstalk due to transversely radiated vibrations [24]. While a measureable MMG signal may be detected both proximal and distal to the muscle belly, the signal features may not have uniform spatial distributions [25-27], thereby affecting the performance of pattern classifiers trained with multi-dimensional feature sets derived from one specific location or sensor configuration. The purported advantage of MMG robustness to sensor placement therefore needs to be re-investigated in a pattern recognition paradigm. A degradation of classification accuracy due to variations in sensor placement would have implications for the design, training and practicality of multifunction MMG switching interfaces.

The amplitude of the MMG signal is known to reach its maximum at the muscle belly and decrease towards the tendon insertions [2,28,29]. Wave propagation theory would suggest that sound waves propagate in all directions away from the source, filtered by soft tissue, thus producing a time-dependent spatial distribution at the surface of the muscle. Multi-channel MMG signals exhibit in-phase vibrations along the longitudinal axis of the muscle fiber and diminished, phase-shifted vibrations transverse to the fiber direction [24,28]. When monitored with a two-dimensional grid of accelerometers, temporal and spectral features of MMG signals elicited during single motor unit activity show substantial spatial dependencies on the muscle surface [26]. The interference MMG signal during voluntary muscle contraction, however, may not always exhibit the spatial dependency of signal features seen during single motor unit activity [30], possibly due to uniform activation maps generated by the summation of heterogeneous activity of multiple motor units [25].

In a pattern recognition-based control framework, a significant spatial dependency of MMG signal features during voluntary contraction would result in a drift in the classifier boundaries in the feature space when the transducer's position is perturbed, thus resulting in a degradation of classification accuracy. However, if the signal features show only weak spatial dependencies, the features may be robust to position variation and thus retain the discriminatory information among multiple classes of muscle activity. The effect of spatial perturbations is dependent on a number of factors, such as the muscle architecture [31], the choice of features [30], feature dimensionality, the number of target classes, and the choice of classifier.

In this study, we test the effect of changes in accelerometer location in a single-site, tri-state classification paradigm. This study attempts to emulate the performance of an MMG classifier trained with the accelerometer in one location and then deployed in a slightly different location. MMG signals were simultaneously recorded by accelerometers located at, and radial to, the belly of the flexor carpi radialis muscle while participants held their hand in the extended, flexed, and semi-pronated positions. The classifier, trained using features from the reference accelerometer (located over the muscle belly) to maximize three-class classification accuracy, was tested with features from the longitudinally and transversely displaced accelerometers. The emphasis of this study is not the classification method per se, but rather the effect of accelerometer displacements in a typical MMG signal classification paradigm. A single MMG channel is therefore used instead of the added information and higher classification accuracy afforded by monitoring multiple muscle sites [20].

## II. Methods

### A. Participants

A convenience sample of twelve able-bodied individuals (4 male), aged 23.5 ± 4 years, provided written consent to participate in the study. Participants were healthy, had intact forearm musculature, and no previous history of musculoskeletal illness. The experimental protocol was approved by the research ethics boards at Bloorview Kids Rehab (#08-056) and University of Toronto, and was in compliance with the Helsinki Declaration and Canada's Tri-council Policy statement on ethical conduct for research involving humans.

### B. Experimental equipment

MMG signals were detected with five uniaxial accelerometers (BU-7135 Knowles Acoustics low-frequency response accelerometer, sensitivity 28 mV/g, linear 2 Hz - 1 kHz, weight 0.28 gm, size 8 × 5.5 × 2 mm). A custom terminal box was built to amplify the accelerometer signals (AMP04, Analog Devices, gain ≈ 100) and interface the accelerometers with a terminal block (National Instruments, BNC-2095). Vibrations of known amplitude were applied to each accelerometer-amplifier assembly via a mechanical shaker (Modal Shop, K2007E01) to ensure uniform gain across all MMG sensors. The MMG signals from the terminal block were channelled through an analog signal conditioning input module (National Instruments, SCXI-1102C), sampled at a rate of 1 KHz (National Instruments, PXI-6052E, 16-bit, ± 5V), and the digitized signals were stored on the controller's hard drive. A custom LabView graphical user interface with a manual trigger was used to start data acquisition and visually cue participants to perform wrist flexions, wrist extensions, and rest (hand held in the semi-prone position).

### C. Experiment Protocol

**Figure 1:**
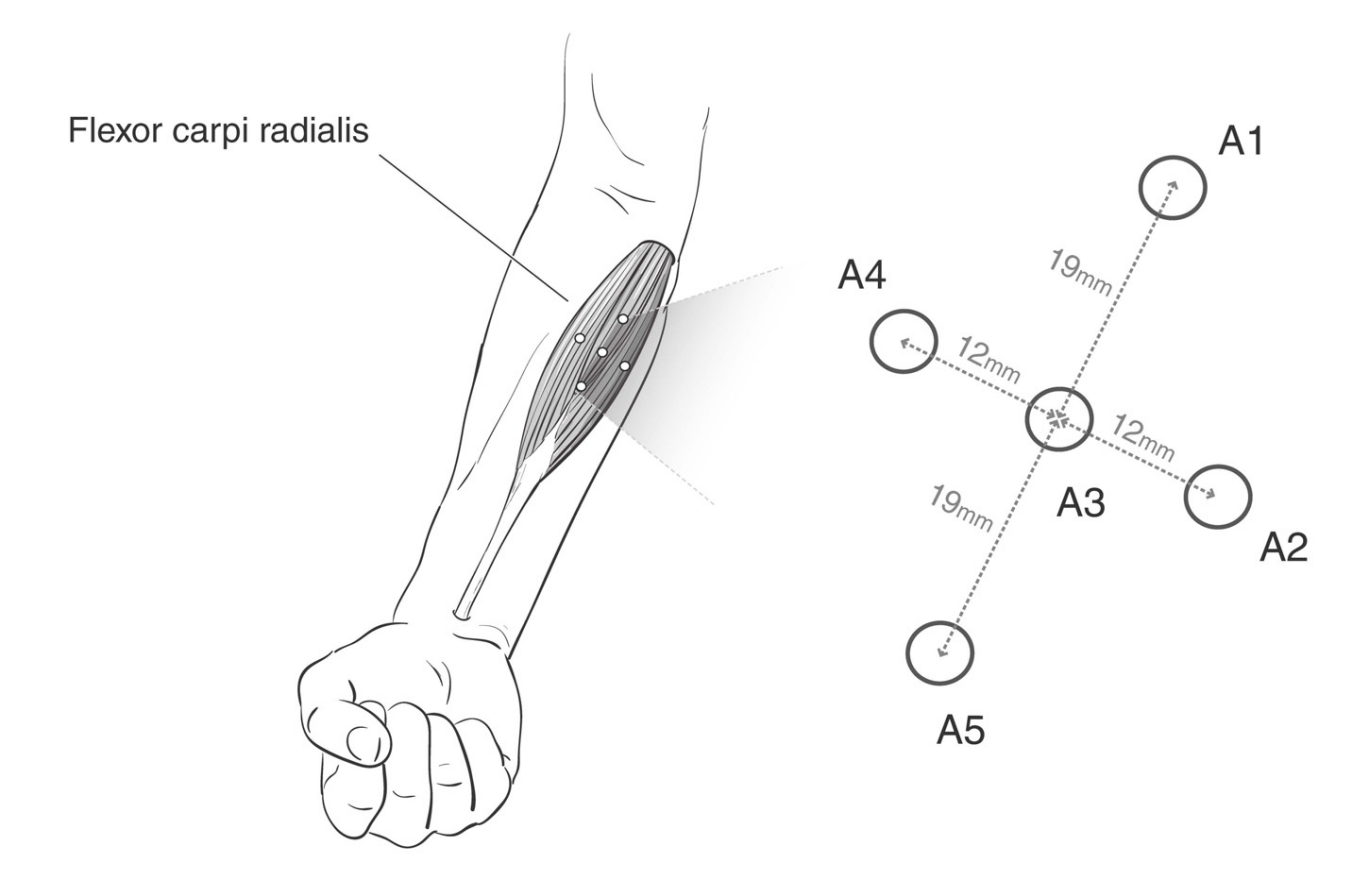
**Schematic representation of the accelerometer locations for MMG recordings**.

Participants were instructed not to perform fatiguing upper-limb exercise twenty-four hours before the trials. The five accelerometers were attached to the skin overlying the flexor carpi radialis (FCR) muscle using 3M™ polyester medical tape. Each accelerometer's sensitive axis was perpendicular to the skin surface. As shown in Figure 1, a reference accelerometer, A3, was positioned at the muscle belly while peripheral accelerometers A1 and A5 were situated along the longitudinal axis of the muscle, and peripheral accelerometers A2 and A4 were secured along the transverse axis. The muscle belly and the longitudinal axis were estimated by palpating the muscle and referencing anatomical landmarks, i.e., the medial epicondyle of the humerus and base of metacarpals [32]. Participants were seated on a chair fitted with a custom arm-rest with u-shaped groves that stabilised the forearm and supported the wrist and elbow. A tri-axis accelerometer (MMA7260Q, Freescale Semiconductor) was affixed to the participant's hand solely for the determination of hand movement times. MMG data were simultaneously recorded from accelerometers A1 to A5 in five trials. In each trial, participants were cued to perform 20 repetitions of a target activity sequence, namely, rest - flexion - rest - extension, where each activity was maintained for 3 seconds.

### D. Signal pre-processing

Accelerometer signals were band-pass filtered with a 5^th ^order Butterworth filter with a pass-band of 5-50 Hz. The low cut-off attenuates the effects of movement [33], while the high cut-off attenuates any noise beyond the accepted MMG signal range. The continuous data streams were spliced into non-overlapping epochs of 256 ms. The known timing of the target activity sequence, together with the timing of the actual hand movements determined by the tri-axis accelerometer, were used to categorize each epoch into one of three classes: rest, flexion and extension.

### E. Correlation between the reference and peripheral accelerometer signals

The cross-correlation function is a measure of the similarity between two signals as a function of the time displacement between them. For two signals *x*_*i *_and *x*_*j*_, the normalized cross-correlation function is given by:(1)

where *N *is the common signal length, *k *is the lag i.e. -*N *<*k *<*N*, *k *∈ *Z*, and *e *is the energy of the signal, evaluated by(2)

*R*_*i,j *_can assume values between [-1, 1], with values close to 1 indicating strong similarity.

We computed the pair-wise cross-correlations at *k *= 0 between the reference signal *x*_3 _and the signals *x*_1_, *x*_2_, *x*_4_, *x*_5_, recorded from accelerometers A1, A2, A4, A5, respectively.

### F. Feature selection

In [20], we proposed an MMG pattern recognition scheme where discriminatory signal features, selected using a genetic algorithm (GA), were classified using a linear discriminant analysis (LDA) classifier. In this study, a similar method was used to determine discriminatory features from a single MMG channel.

A comprehensive set of 70 features were extracted from representations of the accelerometer signals in the time, frequency and time-frequency domains. These features included: ^1^RMS; ^2^signal memory [34]; ^3^mean of absolute amplitude difference; ^4^waveform length; ^5^Wilson's amplitude [35]; ^6^slope-sign change; ^7-11^relative energy relative energy contribution from five wavelet decomposition levels [36]; ^12-18^seventh-order auto-regressive (AR) coefficients; ^19-25^cepstral coefficients derived from the AR coefficients [37]; ^26^log of RMS [38], ^27^median, ^28^mean of absolute value, ^29^mean absolute deviation; ^30^entropy rate [36], ^31^normality, ^32^the stationary test statistic [20]; ^33^number of zero crossings, ^34^minimum and ^35^maximum displacements of the time-domain signal; ^36,37^mean, ^38,39^variance, ^40,41^inter-quartile range, ^42,43^skew, ^44,45^kurtosis and ^46,47^dispersion ratio of the signal and its power spectrum; ^48,49^peak and ^50,51^median frequencies and power at these frequencies; ^52 ^ratio of signal power in the 3-15 Hz range to that in the 15-50 Hz range; and ^53-70^the energy in each of three equal segments of details and approximation, evaluated from the five-level discrete wavelet decomposition of the signal. Subsets of these features have been used for classification of MMG [20] and other physiological signals [35,36,39].

The GA sought participant-specific subsets of *D *= 12 discriminatory features using data from the reference accelerometer, A3, recorded in the first trial only. In a previous MMG classification study, *D *≥ 10 was recommended for adequate signal classification [20]. The optimization criterion for the GA search was the average three-class classification accuracy with an LDA classifier. Using the GA-recommended subsets of features, a subset of 25 features common to all participants, labelled 1-25 above, were selected for MMG classification.

### G. Classification

#### 1) **Linear discriminant analysis**

Let *Q *represent the number of classes and *N*_*q *_the number of samples in the *q*^*th *^class, with *q *= 1,...,*Q*. Consider the *M*-dimensional feature vector , derived from the *i*^*th *^sample of the *q*^*th *^class, where *i *= 1,...,*N*_*q*_. The sample class covariance matrix  is given by(3)

where  is the mean vector of class *q*. The pooled within-class scatter matrix is,(4)

where *p*_*q *_is the a priori probability of class *q*. The between-class scatter matrix measures the dispersion of the class mean vectors about the overall mean vector, and is given by,(5)

where  is the expected vector of the mixture distribution.

LDA projects the feature vectors  onto a lower dimensional space, , *D *≪ *M*, using a linear transformation Θ_*MxD *_which maximizes the ratio of the between-class scatter matrix **S**_*b *_to the pooled within-class scatter matrix **S**_*w*_. Let  denote the *i*^*th *^projected feature vector, from the *q*^*th *^class. The action of LDA can be compactly written as,(6)(7)

where **W **and **V **are matrices formed by horizontally stacking the feature vectors  and , respectively, for all samples *i*. It can be shown that the optimal projection matrix Θ* is the one whose columns are the eigenvectors corresponding to the *D *largest eigenvalues of the eigenvalue decomposition of **S**_*W *_^-1 ^**S**_*B *_[40]. As long as *D *≥ *Q*-1, no information is lost when the classes are normally distributed [41]. In this study, the dimensionality of the feature vectors  was reduced to  by LDA projections.

#### 2) Centre-trained classifier

In each trial, the data from the reference accelerometer, A3, were separated into a training set and a test set using five-fold cross validation. The training set was used to evaluate the optimal LDA projection matrix, Θ*, and train a Bayesian LDA classifier to discriminate among the three classes. This type of training will be termed the "centre-trained" approach given the central location of the reference accelerometer. The test data for each fold *f *of each of the five experimental trials *s *(see Section II.C) were projected by Θ* and classified to generate the classification accuracy for the reference sensor, *C*_3_(*s, f*).

The centre-trained classifier was also was tested separately with MMG signals from each peripheral sensor. This evaluation generated classification accuracies,*C*_i_(*s, f*), for sensors *i *∈{1,2,4,5}, trials *s *∈{1,...,5}, and cross-validation folds *f *∈{1,...,5}. In each case, the projection matrix Θ* and the classifier boundaries were determined using the training data from A3. For each participant, the test accuracies for each accelerometer were pooled across the trials and cross-validation folds, and the distributions of classification accuracies for each accelerometer were compared to that of the reference sensor, i.e., *C*_3_, using rank-sum tests with. *α *= 0.05 In addition, the change in classification accuracy, Δ*C*_i,3_, from the reference (A3) to the *i*^th ^peripheral accelerometer in a given trial and fold was determined as(8)

#### 3) Group-trained classifier

Hargrove et al. [42] proposed that EMG classifiers trained with exemplars of signals recorded at possible displaced locations may mitigate the effect of electrode displacements. To investigate the potential of this approach to benefit single-site MMG classification, a 'group training' method was also implemented. In this case, the training and testing data consisted of subsets of signals from all five accelerometers. The distributions of classification accuracies for each accelerometer using the centre-trained and group-trained methods were compared using rank-sum tests with a significance level *α *= 0.05.

#### 4) Locally-trained classifier

Theoretically, the best performing classifier at a given recording site is one which is trained and deployed with signals arising exclusively from the same site. To assess the value of these 'locally-trained' classifiers [42], and thus, the ability of MMG data from each muscle site to discriminate among multiple activity classes, five separate classifiers were trained, one for each accelerometer location. Classification accuracies were estimated at each site assuming a single-site classifier. Multiple rank-sum tests were used to identify median classification accuracies that were different from that of accelerometer A3, at a Bonferroni adjusted significance level of *α *= 0.0125.

## III Results

**Figure 2:**
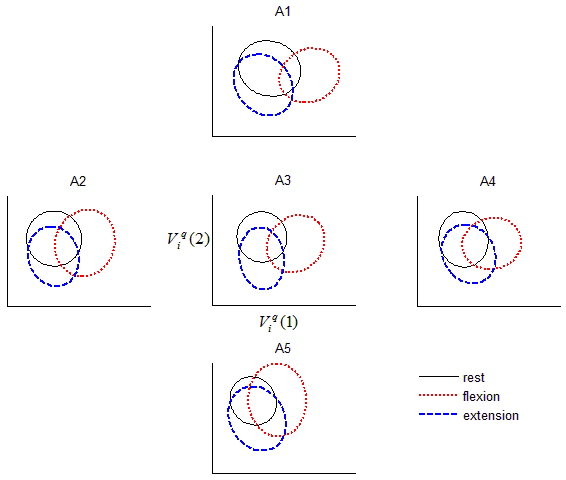
**Effect of accelerometer location on inter-class separation. **The ellipses depict the boundaries within which 95% of the LDA-projected features lie. The projection matrix was optimized for separability using MMG signals recorded from A3. Note the increased overlap among classes once the accelerometer is positioned away from the reference location, A3. Data are shown for participant 4, trial 1.

The mean zero-lag cross-correlation between signals from the peripherally located accelerometers (A1, A2, A4, and A5) and the signal from the reference accelerometer (A3) ranged from 0.72 to 0.96. Substantial degradation in classification accuracy was often seen despite high correlations between peripheral and reference signals, for example, *R*_4,3_(0) = 0.94 and Δ*C*_4,3 _= 64% for participant 10. Conversely, minimal degradation was observed in some instances of high correlation, for example, *R*_5,3_(0) = 0.81 and Δ*C*_5,3 _= 0.9%, again for participant 10. Table 1 summarizes the degradation in accuracy for each peripheral sensor location, for each participant. While there was no consistent pattern of degradation across sensor locations, the A5 location exhibited the mildest degradation (approximately 2%) across participants. Corroborating the above remarks, we observed that the class boundaries in feature space drifted non-systematically as the accelerometer position was changed from the reference location, as exemplified in Figure 2.

**Table 1:** Degradation in classification accuracy across sensor positions

Participant	Accuracy C_3 _(%)	A1-A3	A2-A3	A4-A3	A5-A3
		ΔC_1,3_	ΔC_2,3_	ΔC_4,3_	ΔC_5,3_
1	69.8 ± 4.3	**7.5 **± **4**	**6.5 **± **4**	**6.8 **± **3**	1.9 ± 3
2	71.0 ± 5.9	4.0 ± 4	**6.9 **± **5**	1.5 ± 5	2.0 ± 4
3	69.6 ± 4.5	**5.7 **± **4**	**6.6 **± **4**	**7.7 **± **5**	**7.4 **± **5**
4	74.6 ± 2.7	**11.4 **± **4**	*4.7 ± 4*	*10.0 *±	**7.5 **± **4**
5	75.1 ± 4.2	*11.8 ± 4*	*12.9 ± 4*	1.7 ± 3	**4.5 **± **4**
6	69.1 ± 3.9	**7.2 **± **5**	*5.7 ± 4*	**9.1 **± **5**	1.5 ± 4
7	76.2 ± 4.3	**14.9 **± **3**	*17.4 ± 3*	**4.6 **± **5**	2.1 ± 4
8	71.2 ± 4.4	2.0 ± 3	0.8 ± 2	**11.1 **± **5**	3.6 ± 5
9	73.1 ± 3.0	**5.9 **± **3**	**5.6 **± **3**	**7.2 **± **4**	1.0 ± 3
10	76.4 ± 3.4	**7.7 **± **4**	1.0 ± 4	**6.4 **± **3**	0.9 ± 3
11	76.8 ± 3.0	1.4 ± 3	1.7 ± 2	**4.9 **± **3**	1.7 ± 3
12	69.7 ± 3.9	*14.1 ± 5*	**12.2 **± **2**	**4.6 **± **3**	**4.9 **± **3**
					
Avg	72.7 ± 0.6	**7.8 **± **5**	**6.8 **± **6**	**6.3 **± **4**	**3.0 **± **4**

Values shown are mean ± standard deviation across all trials. Bold and italic values indicate that C_*i *_and C_3 _are significantly different (p < 0.05). Italic values indicate that the accuracy of the locally-trained classifier at this location was lower than that of A3, suggesting that this recording site yields signals that poorly reflect FCR activity.

**Figure 3:**
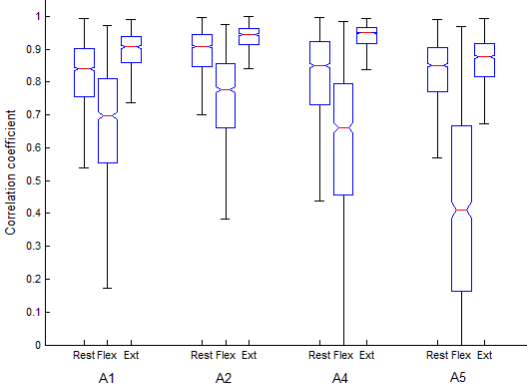
**Typical distributions of the zero-lag cross-correlation between the reference (A3) and peripheral sensor locations (A1, A2, A4 and A5) as a function of muscle activation class. **Data are shown for participant 4, pooled across all trials.

There was an effect of contraction type on the cross-correlation between the reference and peripheral sensors (Kruskal-Wallis test, p < 0.05). As an example, Figure 3 depicts the distributions of the cross-correlation coefficients for different contractions and sensor locations, for participant 4. Note the lower cross-correlation during flexion at all peripheral sensor locations. This same pattern was observed in ten of the 12 participants.

**Figure 4:**
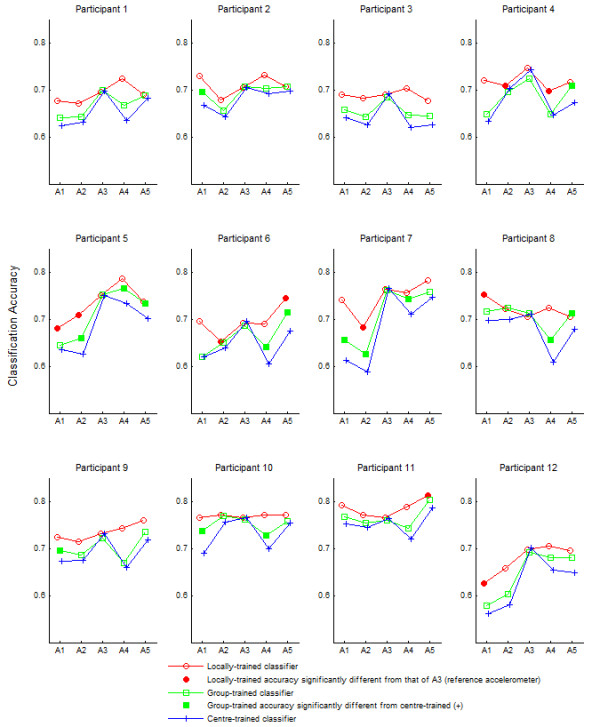
**Classification accuracy for each participant as a function of accelerometer location and training method. **The shaded squares denote group-trained accuracies that are significantly different from those obtained for the centre-trained classifier. The shaded circles denote locally-trained accuracies that are significantly different from that of A3.

Figure 4 shows the classification accuracies for each participant for the three classifier training methods: centre-trained, group-trained and locally-trained. Unsurprisingly, the locally trained classifiers generally exhibited the highest accuracies, followed by the group-trained classifiers and finally the centre-trained classifiers with the lowest accuracies. The shaded circles indicate instances where the locally-trained classification accuracy at a particular site was statistically different from that of A3. In some instances, the locally trained classifier at a peripheral site performed more poorly than A3 (e.g., sensor A2 and A4 for Participant 4), suggesting that certain sites are inherently less suited to reflect flexor activity. On average, sensors A3 and A5, both situated along the longitudinal axis of the muscle, showed the highest locally-trained classification accuracies. The shaded squares in Figure 4 indicate instances where group-trained classification accuracies were statistically different from the centre-trained accuracies, thus demonstrating the potential benefit of training the MMG classifier with exemplars from displaced locations.

## IV. Discussion

Spatial dependencies of temporal and spectral MMG features have been previously noted but not systematically quantified in terms of their impact on classification [25-27,29]. From Table 1 and Figure 4, it is evident that the accuracy of centre-trained MMG pattern classifiers usually degrades when the accelerometers are displaced from the location over which the classifier was trained, suggesting that signal features representing different muscle activity states have drifted (e.g., Figure 2), thus weakening the efficacy of the original classification boundaries. This lack of spatial robustness is due to the fact that the centre-trained classifier has no internal representation of signals from other spatial sites.

Once the classifier is exposed to signals from other sites during training as in the group training scenario, greater robustness to displacement was observed. In this case, the classifier has an opportunity to form a shared internal representation of signals from all sites during training. The improved accuracy suggests that group-training may practically mitigate some of the negative effects of accelerometer displacement. Finally, in the local training paradigm, multiple classifiers are created, each with a dedicated representation of signals from a specific muscle site. Given that these location-specific classifiers were only exposed to signals from their own location, accuracies exceeded those of group and centre-trained classifiers. However, from a practical standpoint, this approach incurs the greatest representational cost.

In a traditional pattern-recognition framework, multiple muscle sites are typically probed [17,20]. For example, using the same experimental protocol, when the data set included features simultaneously extracted from the FCR muscle (accelerometer A3) and the belly of the extensor carpi radialis longus (ECRL) muscle, the average classification accuracy was 91 ± 3% (3 classes, continuous classification). Nonetheless, we elected to focus on a single-site tri-state classification paradigm to ensure an observable effect of accelerometer position on classification. The classification accuracy was modest when trained with signals from a single-site (≈73% for 3 classes, continuous classification) and thus the classifiers would likely be susceptible to changes in accelerometer placement. Likewise, in studies of EMG-based control, classifier degradation due to displacements were more pronounced when fewer muscle sites were used [43,44].

The FCR muscle is a long fusiform muscle [32] with an expanded belly and the muscle fibres arranged more or less parallel to each other and the long axis of the muscle. The mechanomyogram is known to show in-phase vibrations along the longitudinal axis of the muscle fiber, and a decrease in amplitude and changes in phase transverse to the fiber direction [24,28]. We therefore expected that classification accuracy would be less affected by displacements along the longitudinal axis [26]. This was not apparent for all participants, perhaps due to limitations in approximating the long axis of the muscle. In addition, the correlation coefficients along the longitudinal axis (*R*_1,3 _and *R*_5,3_) were generally not higher than those along the transverse axis (*R*_2,3 _and *R*_4,3_). Accuracy degradation, however, was lowest for A5 - a site along the muscle axis but closer to the tendon.

The cross-correlation coefficient, a measure of temporal similarity, was not always a good predictor of the effect of displacements on classification accuracy. Like many biosignals (eg., electromyograms [45], electroencephalograms [46]), MMG may exhibit non-linearities [47]. While interdependence among the signals may be better analysed with information theoretic cross-entropy measures [48], non-linear analyses are deferred for future studies. It is interesting to note that, for most participants, the cross-correlation between A3 and the peripherally located sensors was lowest during flexion (Figure 3). During voluntary contraction, the mechanical activities of motor units are asynchronous, and motor units fire at different rates [4]. MMG signal features, such as peak-to-peak value, are dependent on the location of the active motor unit with respect to the accelerometer position [25]. These asynchronous localized increases in motor unit activities of the FCR during flexion likely caused the accelerometer signals to de-correlate. This has two important implications for pattern recognition of MMG. First, some classes of muscle activity may be more affected by accelerometer displacements than others. The activity class for which the muscle under consideration serves as the main driver appears to be most gravely affected. Second, because of the task-specific dependence, multi-site cross-correlation coefficients may themselves serve as discriminatory features for multifunction classification.

As seen in the distribution of accuracies for the locally trained classifiers (Figure 4), some sites around the FCR were occasionally more discriminatory than others. During single motor-unit activation, the muscle generates acceleration waves that propagate transversely over long distances [24]. The MMG signal is not specific to the activity of the underlying muscle when several muscles are collectively active. The amount of cross talk, and hence, classification accuracy at each site, may be dependent on its proximity to other forearm muscles that assist with wrist flexion, extension and semi-pronation (rest).

While accelerometers are the most commonly used transducer for MMG recording, MMG may also be recorded by microphones [9,49,50], piezoelectric contact sensors [10,11] and laser distance sensors [13]. A number of studies have compared the different transducers for detecting MMG [11,50,51]. Each transducer, though measuring the same phenomenon, may have signals with different physical units, may introduce different loading pressure and mechanical discontinuities on the skin, and may have different sensitivities to muscle vibrations and motion artefact. Further, the area in contact with the skin, or, for non-contact laser sensors, the area investigated by each transducer differs. Consequently, the transducer used to record MMG may have implications for the signal features used for classification, the spatial dependencies of the features, and the effect of transducer displacements on classification accuracy.

The extent of classification degradation due to sensor displacements is likely influenced by a number of factors, such as the number of muscle sites from which signals are acquired, the number of target classes, selected signal features, complexity of the classifiers, training method employed, muscle architecture, and the transducer used to record MMG. Our results suggest that it is worthwhile to carefully consider sensor attachment gear which may offer consistent placement of the sensor on the skin surface when designing multifunction MMG switching interfaces.

## V. Conclusion

Single-site forearm MMG signal feature values vary non-systematically with spatial location. Accelerometer displacements of only 1-2 cm from the site providing training data can cause sufficient feature drift to significantly diminish classification accuracy. This finding emphasizes the importance of consistent sensor placement between MMG classifier training and deployment for accurate control of MMG switching interfaces. Classifier training strategies which involve MMG signals from several displaced locations may enhance classifier robustness to variations in sensor position.

## Competing interests

The authors declare that they have no competing interests.

## Authors' contributions

NA conceived the study, designed the experiments, analyzed the data, and drafted the manuscript. TC was the primary supervisor of the study. He advised on the design of the experiments, advised on data analysis, and edited the manuscript. BS assisted with study design, data collection and data analysis. ES advised on data analysis and edited the manuscript. All authors read and approved the final version of the manuscript.
